# Valorization of *Ulva prolifera* Biomass into Biostimulants: Effects of Extraction Processes on Cherry Radish Growth, Nutritional Quality and Soil Fertility

**DOI:** 10.3390/plants15192978

**Published:** 2026-09-29

**Authors:** Hailong Wu, Wenli Jiang, Wen Liu, He Li, Sufang Li, Fei Xu, Fengrong Tian, Xinxing Shi, Baolin Yuan

**Affiliations:** 1 Jiangsu Key Laboratory of Marine Bioresources and Environment, Jiangsu Institute of Marine Resources Development, Jiangsu Ocean University, Lianyungang 222005, China; 2 State Key Laboratory of Marine Environmental Science, College of Ocean and Earth Sciences, Xiamen University, Xiamen 361005, China; 3 Jiangsu Zhonglian Agricultural Development Co., Ltd., Lianyungang 222023, China; 4 China Zhonglan Lianhai Design and Research Institute Co., Ltd., Lianyungang 222004, China; 5 Lianyungang Agricultural and Commercial Holding Group Co., Ltd., Lianyungang 222006, China

**Keywords:** enzymatic hydrolysis, microbial fermentation, cherry radish, biostimulant, soil improvement, sustainable agriculture

## Abstract

Frequent green tides of *Ulva prolifera* in China’s Yellow Sea generate massive algal biomass that pollutes coastal environments and disrupts aquaculture. Valorizing this waste into biofertilizers offers a sustainable strategy for marine ecological remediation and agricultural production. This study compared the effects of *U. prolifera* extracts prepared via enzymatic hydrolysis (UE) and enzyme-coupled microbial fermentation (UEF) on cherry radish growth, nutritional quality, and soil fertility across six concentrations (5–30%). Both extracts exhibited a typical biphasic “low-promotion, high-inhibition” dose–response pattern. UEF at 5–10% significantly promoted root development, with maximum root weight (5.1 g/plant) at 10% and diameter (1.7 cm) at 5%. UE at 15–20% achieved root weight comparable to the Hoagland solution (HO), but with lower peak values than UEF. At equivalent concentrations, UEF generally outperformed UE in enhancing nutritional quality—maximizing anthocyanins (4.8 mg/g FW), vitamin C (5.2 mg/g FW), soluble proteins (13.5 mg BSA/g DW), and soluble sugars (17.0 mg glucose/g DW)—as well as soil fertility (N/P/K contents). Both extracts lowered soil pH by 0.5–0.6 units (converging to ~7.1 after 40 days), suggesting their potential for saline–alkali soil improvement. These findings suggest that *U. prolifera* extracts, particularly those prepared via enzyme-coupled fermentation, may serve as promising dual-function biostimulants and soil conditioners. The optimal concentrations (5–10% for UEF; 15–20% for UE) provide practical guidance for field application, offering a “waste-to-value” solution that simultaneously mitigates green tide hazards and supplies sustainable agricultural inputs, thereby contributing to both marine ecological restoration and sustainable crop production.

## 1. Introduction

As an emerging biostimulant and biofertilizer, seaweed extracts have been demonstrated to enhance crop productivity, stress tolerance, and quality [1,2]. On one hand, seaweed extracts are rich in bioactive compounds, including polysaccharides, betaine, mannitol, essential nutrients (e.g., N, P, K, Fe, Zn), and phytohormones (e.g., cytokinins, auxins). These bioactive substances regulate plant physiological metabolism, improve photosynthetic efficiency, activate the antioxidant defense system, and trigger plant stress responses [3,4]. On the other hand, exogenous application of seaweed extracts to soil supplies carbon sources and nutrients to rhizosphere microorganisms. This process may effectively activate beneficial microbial communities, accelerate the mineralization and cycling of soil nutrients, and ultimately ameliorate soil physicochemical properties and further elevate soil fertility [5,6].

The growth-promoting and soil-enhancing effects of seaweed extracts in crop production are well documented. For instance, foliar spraying of *Kappaphycus alvarezii* and *Gracilaria edulis* extracts on potato plants has been shown to significantly increase tuber weight, tuber bulking rate, and overall yield, while also improving tuber quality (vitamin C and reducing sugar content) [7]. Similarly, in onion, seaweed extract application enhanced vegetative growth (leaf number, length, width, and plant height), reduced plant mortality, and improved multiple bulb quality traits, including stem diameter, equatorial and polar diameters, ring count, and total soluble solids [8]. Er-rqaibi et al. [5] also reported that under drought stress, the crude extract (5%) from the brown alga *Fucus spiralis* increased radish fresh weight by 47.43% and decreased leaf proline content by 23.45% relative to the control group. In addition, long-term seaweed fertilizer application has been reported to enrich soil with polysaccharide-degrading and diazotrophic bacteria (e.g., Acidobacteria, Cyanobacteria, and *Methylobacterium*), thereby optimizing the soil microecology and improving fertilizer use efficiency [9].

The extraction and preparation technologies may affect the biological efficacy and functional activity of seaweed extracts. Chemical hydrolysis and physical extraction, though widely used, suffer from degradation of thermosensitive compounds and high energy/cost inputs [10,11]. Biological approaches, particularly enzymatic hydrolysis and microbial fermentation, have garnered increasing attention due to their mild reaction conditions and superior retention of bioactive constituents [12,13,14,15,16]. Enzymatic hydrolysis employs specific enzymes such as cellulases, polysaccharide lyases and proteases to degrade algal macromolecules into bioactive oligosaccharides and peptides, while simultaneously facilitating the release of mannitol, phenolic compounds, and phytohormones [13,17]. These metabolites collectively regulate plant metabolism (e.g., photosynthesis, antioxidant systems, osmoregulation, hormone signaling) to promote growth, quality, and stress resistance. Microbial fermentation, alternatively, utilizes beneficial strains such as *Bacillus subtilis*, *Paenibacillus polymyxa*, and *Trichoderma* sp. to metabolically degrade algal biomass, releasing endogenous bioactives while generating de novo metabolites (e.g., oligosaccharides, organic acids, phenols, and auxin analogs) that can promote plant germination, growth, and abiotic stress tolerance [1,12,16,18].

The combined enzymatic-fermentation process, whether sequential or simultaneous treatment, is expected to integrate the benefits of both methods, enabling efficient bioactive release and potentially conferring new functionalities through microbial biotransformation. Nevertheless, comparative studies on the physiological and metabolic effects of single enzymatic versus enzymatic-fermentation coupled extracts on crops remain scarce. Furthermore, the concentration of seaweed extract is another pivotal factor modulating its biological activity: appropriate concentrations promote plant growth, whereas excessive concentrations may inhibit plant growth through osmotic stress or component antagonism [8,15]. Therefore, investigating the concentration-dependent responses of crop growth and product quality to seaweed extracts prepared via different processes is of considerable practical significance for the high-value utilization of seaweed as an agricultural input.

*Ulva prolifera*, the primary causative species of green tide disasters in the Yellow Sea of China, poses a serious threat to marine ecosystems and coastal economies through its massive outbreaks [19,20]. In contrast, this alga is rich in polysaccharides, proteins, minerals, and plant growth regulators, rendering it a promising renewable biomass resource with considerable agricultural potential [21,22]. The conversion of *U. prolifera* biomass into crop cultivation inputs not only aligns with circular economy principles but also provides an eco-friendly strategy for mitigating green tide hazards at the source. Although seaweed extracts have been extensively documented to improve crop growth, stress tolerance, and product quality, studies on the growth-promoting effects of *Ulva*-derived extracts specifically in vegetables remain underexplored [15,23,24].

Cherry radish (*Raphanus sativus* var. radicula) is a small cherry-shaped vegetable belonging to the Brassicaceae family. The roots are abundant in antioxidants and health-promoting phytochemicals (anthocyanins, vitamin C, glucosinolates, polyphenols, etc.), which confer this vegetable with considerable nutritional and medicinal values. To explore the agricultural potential of *Ulva prolifera* biomass, this study used cherry radish as the test material to compare the effects of two extract preparation processes, single enzymatic hydrolysis (UE) and enzyme-coupled microbial fermentation (UEF), across six concentrations (5%, 10%, 15%, 20%, 25%, and 30%) on plant growth (plant height, plant weight, root diameter, root weight), physiological status (photosynthetic pigment content), nutritional quality (anthocyanins, vitamin C, soluble proteins, soluble polysaccharides), and soil physicochemical properties (N, P, K contents, pH, and EC). The findings are expected to elucidate the applicability of different seaweed extraction processes in vegetable production from the perspectives of agronomic traits and physiological metabolic responses, thereby providing a scientific basis for process selection and concentration optimization in the high-value agricultural utilization of green-tide algae.

## 2. Materials and Methods

### 2.1. Preparation of Seaweed Extracts

#### 2.1.1. Seaweed Collection

The green macroalga *Ulva prolifera* used in this study was collected from Binhai New District, Fenghua District, Ningbo City, Zhejiang Province, China (29°07′ N, 121°41′ E). Fresh algal thalli were immediately rinsed and scrubbed with sterile seawater to remove sediment and epiphytes. The cleaned samples were dried at 85 °C for 24 h, and then ground into powder using a grinder and passed through an 80-mesh sieve.

#### 2.1.2. Preparation of *U. prolifera* Extract via Enzymatic Hydrolysis

**Figure 1:**
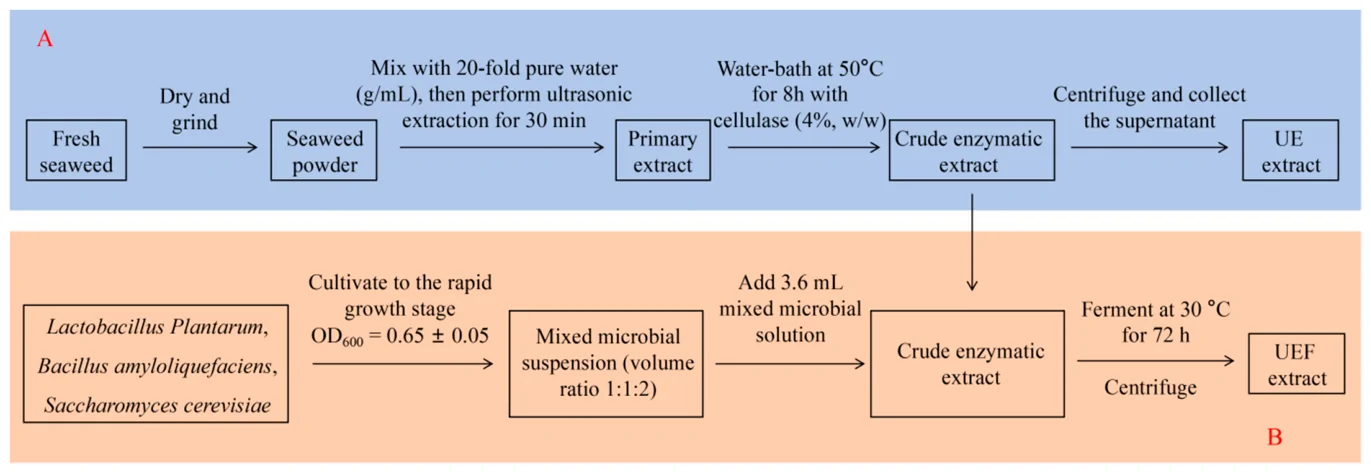
Schematic flowchart of the preparation processes for *U. prolifera* extracts: (**A**) production of *U. prolifera* extract by single enzymatic hydrolysis; (**B**) production of *U. prolifera* extract via combined enzymolysis and microbial fermentation.

Figure 1A illustrates the flowchart for preparing the enzymatic hydrolysate of *U. prolifera* extract (UE). Briefly, *U. prolifera* powder was mixed with distilled water at a ratio of 1:20 (g/mL) and subjected to ultrasonic treatment at 450 W for 30 min for initial extraction. Subsequently, cellulase (Sigma-Aldrich, St. Louis, MO, USA) was added at 4% (*w*/*w*, relative to the dry weight of seaweed powder), and the mixture was incubated in a 50 °C water bath for 8 h, with manual stirring every 45 min to prevent sedimentation. After hydrolysis, the enzyme was inactivated by heating the mixture in a 95 °C water bath for 10 min. The hydrolysate was then transferred to 50 mL centrifuge tubes and centrifuged at 10,000 rpm for 15 min. The supernatant was collected as the UE extract and stored at 4 °C.

#### 2.1.3. Preparation of *U. prolifera* Extract via Enzymolysis-Coupled Microbial Fermentation

To prepare the fermented extract of *U. prolifera*, three microbial strains—*Lactiplantibacillus plantarum* L13, *Bacillus amyloliquefaciens* MMB-02, and *Saccharomyces cerevisiae* A8—were used as described in our previous study [15]. The microbes all obtained from the Key Laboratory of Marine Bioresources and Environment, Jiangsu Ocean University. Table S1_supplementary data outlines the basis for strain selection and the potential contributions of each microorganism to the fermentation process. Each strain was separately cultured in MRS, LB, and PDA liquid media, respectively, until the mid-logarithmic phase (optical density OD_600_ = 0.65 ± 0.05).

Figure 1B illustrates the flowchart for preparing the *U. prolifera* extract via enzymolysis-coupled microbial fermentation (UEF). After the 8 h enzymatic hydrolysis, 3.6 mL of mixed microbial culture (containing *L. plantarum*, *B. amyloliquefaciens*, and *S. cerevisiae* at a volume ratio of 1:1:2) was added to the crude enzymatic extract. The mixture was then incubated in a dark incubator at 30 °C for 72 h. The fermented broth was centrifuged at 10,000 rpm for 15 min, and the supernatant was collected as the UEF extract and then stored at 4 °C.

Before the experiments, six working concentrations (5%, 10%, 15%, 20%, 25%, and 30% dilution of the stock UE and UEF extract, *v*/*v*) were prepared by adding different volumes of distilled water.

### 2.2. Seed Disinfection and Germination of Cherry Radish

Cherry radish seeds were purchased from the Beijing Academy of Agricultural Sciences. Plump and high-vigor seeds were selected. They were soaked in distilled water at 40 °C for 10 min, and then disinfected with 2% sodium hypochlorite solution for 2 min. After thorough rinsing with distilled water, the seeds were placed in germination boxes. The bottom of each box was lined with moist cotton pads; seeds were evenly spread on the pads and covered with another layer of moist cotton pads. After incubation in the dark at 25 °C for one day, robust and uniformly growing seedlings were selected and transferred to seedling pots (20 cm in diameter). The potting soil was collected from Longxiaozai Colorful Countryside (34°37′ N, 119°20′ E), Haizhou District, Lianyungang City, Jiangsu Province. The unfertilized soil had an initial pH of 7.8–8.2 and an electrical conductivity (EC) of 1.66–1.87 mS/cm, classifying it as slightly-to-moderately saline–alkali soil.

### 2.3. Cultivation and Fertilization of Cherry Radish

Cherry Radish was cultivated under controlled laboratory conditions in intelligent low-temperature light incubators (Model ZGX-600C, Kesheng Experimental Instrument Co., Ltd., Ningbo, China) from August to November 2025. The culture conditions were set as follows: a photoperiod of 12 h (light)/12 h (dark), temperatures of 25 °C during the light period and 18 °C during the dark period, and a photosynthetic photon flux density (PPFD) of approximately 108 μmol photons/(m^2^·s). The crops were cultivated for 40 days and evenly irrigated with 100 mL of UE or UEF extracts every 3 days, with commercial liquid Hoagland fertilizer (HO [25]; Leagene, Beijing, China) used as the positive control and pure water (WT) as the negative control. For each treatment, 60 radish seedlings were uniformly planted in 6 seedling pots at a density of 10 seedlings per pot.

### 2.4. Analysis

#### 2.4.1. Growth Traits

At day 40, the crops were gently removed from seedling pots to avoid root injury, followed by soft washing of residual rhizosphere soil with tap water. Vegetative growth traits (plant height, total fresh weight (FW), root fresh weight and root diameter) were quantified to compare the growth of cherry radishes treated with UE and UEF extracts at various concentrations.

#### 2.4.2. Leaf Pigment Contents

Chlorophyll (including chlorophyll *a* (Chl *a*) and chlorophyll *b* (Chl *b*) and carotenoid contents in leaves were quantified following the method described by Lichtenthaler [26]. Briefly, 0.2 g fresh leaf tissue was ground with 1 mL pre-cooled acetone in a pre-chilled mortar and pestle. The homogenized paste was further mixed with an additional 4 mL acetone and centrifuged at 3000 rpm for 15 min. The absorbance of the resulting supernatant was determined at wavelengths of 665 nm, 652 nm and 470 nm using a UV-1800 spectrophotometer (Shimadzu, Kyoto, Japan). Chl *a*, Chl *b* and Car contents (mg/mL) were estimated using Equations (1)–(3):

*C*h*l a* = 16.82 × *A*_665_ − 9.28 × *A*_652_ (1)

*C*h*l b* = 36.92 × *A*_652_ − 16.54 × *A*_665_ (2)

*Car* = (1000 × *A*_470_ − 1.91 × *C*h*l*
*a* − 95.15 × *C*h*l*
*b*)/225 (3)

#### 2.4.3. Root Anthocyanin Content

In this study, anthocyanin content in cherry radish was expressed as cyanidin-3-glucoside (C3G) equivalent, as it is not only the most widely distributed anthocyanin in nature [27] but also one of the predominant anthocyanin components in red radish [28]. Because C3G has a characteristic peak at around 508 nm, the anthocyanin content in roots can be estimated using the spectrophotometric pH difference method [28,29].

Fresh root tissue (0.5 g) was placed in a bead tube and homogenized with 0.5 mL of pre-cooled 90% aqueous methanol using a Precellys 24 Touch homogenizer (Bertin Technologies, Fontaine, France, 5000 rpm, 45 s, 3 cycles). After rinsing with another 4 mL of 90% aqueous methanol, the combined homogenates were transferred to a 15 mL brown centrifuge tube and mixed on an RH-18+3D rotary mixer (Hangzhou Miu Instruments Co., Ltd., Hangzhou, China) at 40 rpm for 60 min. The homogenate was centrifuged at 1000 rpm for 10 min. The supernatant was then filtered through a 0.45 μm Advantec filter (Milpitas, CA, USA) paper, and the filtrate was brought to a final volume of 10 mL with 90% methanol.

Here, two buffer solutions were prepared: 0.2 M KCl–HCl (pH 1.00) and 0.2 M NaAc–HAc (pH 4.50), and their pH values were adjusted with concentrated HCl. Aliquots of the sample extract (200 µL) were mixed with 1.8 mL of the respective buffer solutions and left to stand in the dark at room temperature for 15 min. Absorbance readings at 508 nm and 700 nm were recorded against distilled water as a blank, and the total anthocyanin content (mg/g FW) was calculated according to Equation (4) [28]:

Anthocyanin content = ∆A × M_v_ × f × V/(ε × m) (4)

where $∆A={\left(A_{508}-A_{700}\right)}_{\mathrm{pH}=1.00}-{\left(A_{508}-A_{700}\right)}_{\mathrm{pH}=4.50}$, *M_v_* and *ε* are the molecular weight (449.38 g/mol) and the molar extinction coefficient (26,900/(M·cm)) for C3G, respectively; *f* represents the dilution factor, *V* is the volume of the extraction solution (10 mL); and *m* stands for the mass of the sample (0.5 g).

#### 2.4.4. Root Vitamin C Content

Root vitamin C content was determined using the 2,6-dichlorophenol indophenol (DCPIP) titration method [30,31]. Briefly, fresh fleshy roots (0.8 g) were ground with 10 mL of metaphosphoric–acetic acid solution (HPO_3_-CH_3_COOH, extracting solution) using a mortar. The extracting solution was prepared by dissolving 15 g of HPO_3_ and 40 mL of CH_3_COOH in 200 mL of distilled water and then diluting to 500 mL. After vacuum filtration of the homogenate, the filtrate was collected in a 50 mL volumetric flask. The residue was repeatedly rinsed with the HPO_3_-CH_3_COOH solution, and the rinsates were combined with the filtrate before being made up to a final volume of 50 mL with the same solvent. An aliquot of the extract (10 mL) was then transferred to a 50 mL Erlenmeyer flask and immediately titrated with DCPIP solution until a pink coloration persisted ≥ 5 s. The DCPIP solution was prepared by dissolving 50 mg of 2,6-dichlorophenol indophenol sodium salt in 50 mL of distilled water containing 42 mg NaHCO_3_, with shaking and dilution to 200 mL. The volume of DCPIP consumed was recorded as *V*_1_. A blank titration was performed using 10 mL of the HPO_3_-CH_3_COOH solution, and the titrant volume was recorded as *V*_2_. The DCPIP solution was standardized daily against a freshly prepared ascorbic acid standard solution to determine the titration factor (T, mg/mL). The vitamin C content (mg/g FW) was calculated using the following equation:

*Vitamin C content* = (*V*_1_ − *V*_2_) × *T* × *V*/(*M* × *V*_3_) (5)

where V is the total volume of the sample extract (50 mL), M is the fresh weight of the sample (0.8 g), and *V*_3_ is the aliquot volume used for titration (10 mL).

#### 2.4.5. Root Soluble Sugar and Protein Contents

Cherry radish roots were cut into small pieces and dried at 85 °C to constant weight. A 0.5 g portion of the dried material was homogenized with 10 mL of phosphate buffer (pH 7.4) in a mortar, and the resulting homogenate was employed for the analysis of soluble sugar and soluble protein contents.

Soluble sugar (SS) content was determined by the anthrone method [32]. Additionally, 0.2 mL of the homogenate was combined with 1 mL of freshly prepared anthrone reagent (0.2 g anthrone in 100 mL concentrated H_2_SO_4_), heated in a boiling water bath at 100 °C for 10 min, and centrifuged at 10,000 rpm for 10 min. The absorbance of the supernatant was read at 630 nm. Soluble sugar content was calculated from a glucose standard curve (y = 0.0072x + 0.0092, R^2^ = 0.9991) and expressed as mg glucose equivalents per gram dry weight (mg glucose/g DW).

Soluble protein (SP) content was assayed using the Bradford method [33]. Briefly, the above homogenate was centrifuged at 4500 rpm for 10 min. The resulting supernatant (1 mL) was mixed with 1 mL of Bradford reagent, and after thorough mixing, the absorbance was recorded at 595 nm. Soluble protein content was determined from a standard curve (y = 0.0019x + 0.4164, R^2^ = 0.9989) constructed with bovine serum albumin (BSA) and expressed as mg BSA/g DW.

#### 2.4.6. Soil Physicochemical Property

At day 40, the plants were uprooted and the soil was thoroughly mixed. The collected soil was then analyzed for available N, available P and available K (expressed per kg fresh soil), electrical conductivity (EC), and pH using a calibrated YGY-TRY portable soil analyzer (Wuhan Chenyun Technology Co., Ltd., Wuhan, China) [15].

### 2.5. Data Analysis

All experiments were carried out with six independent biological replicates per treatment (*n* = 6), and for the two control groups (WT and HO), each was tested twice—once alongside the UE treatments and once alongside the UEF treatments—yielding 12 pots in total for each control (*n* = 12). Results were presented as mean ± standard deviation (SD). Statistical evaluation was performed using IBM SPSS Statistics 26.0 (SPSS Inc., Chicago, IL, USA). Two-way ANOVA was used to assess the effects of *Ulva* extract concentration, extract type (UE and UEF), and their interaction on plant growth traits (plant height, plant weight, root diameter, and root weight), nutritional quality parameters (leaf pigments, root anthocyanin, vitamin C, soluble sugar, and soluble protein contents), and soil properties (N, P, K, EC, and pH), with partial η^2^ reported as a measure of effect size. When the interaction term was significant, simple effects analysis was performed: one-way ANOVA followed by Duncan’s multiple range test was used to compare concentrations within each extract type, and independent-sample *t*-tests were used to compare UE and UEF at each concentration. To control the false discovery rate arising from multiple comparisons, the Benjamini–Hochberg procedure was applied to all pairwise *t*-tests within each response variable. Statistical significance was set at *p* < 0.05, with *p* < 0.01 and *p* < 0.001 considered highly and extremely significant, respectively.

## 3. Results

### 3.1. Plant Growth Traits

**Figure 2:**
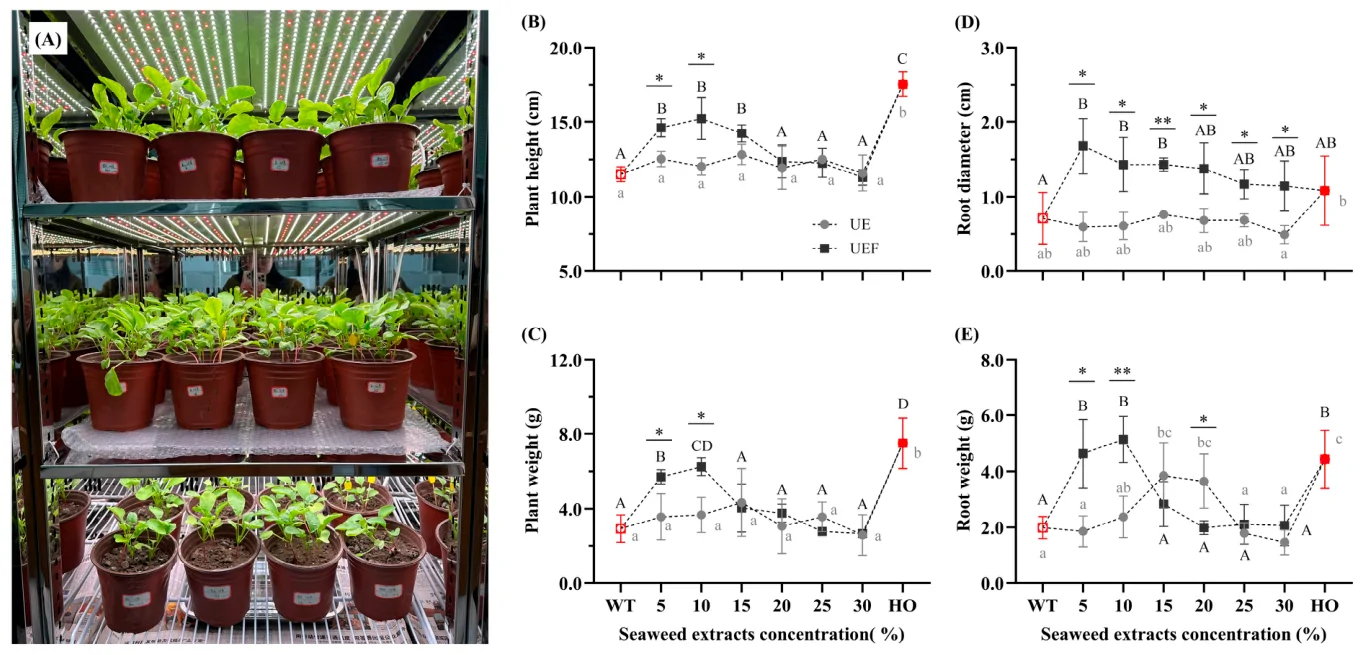
Effects of *U. prolifera* extracts on the vegetative growth of cherry radish: (**A**) cherry radish planting; (**B**) plant height; (**C**) plant weight; (**D**) root diameter; (**E**) root weight. UE and UEF represent the extracts of *U. prolifera* prepared via enzymatic hydrolysis and coupled enzymatic hydrolysis-microbial fermentation, respectively. Different uppercase letters indicate significant differences among UEF treatments, and different lowercase letters indicate significant differences among UE treatments (Duncan’s test, *p* < 0.05). Asterisks indicate significant differences between UE and UEF at the same concentration (independent-sample *t*-test with Benjamini–Hochberg FDR correction: *, *p* < 0.05; **, *p* < 0.01). WT and HO represent the treatment groups irrigated with pure water (red open circles/squares) and Hoagland nutrient solution (red solid circles/squares), respectively).

Figure 2A shows cherry radish plants grown in the incubator under controlled conditions. According to two-way ANOVA results (Table S2_supplementary data), plant height and plant weight were significantly affected by extract concentration, extract type, and their interaction (*p* < 0.05). Because the interaction terms were significant, comparisons were performed at each concentration rather than focusing solely on main effects. At low concentrations (5–15% for plant height and 5–10% for biomass), UEF significantly increased radish above-ground growth compared with the WT control *(p* < 0.05; Figure 2B,C), whereas this growth-promoting effect was abolished at higher UEF concentrations. At low concentrations, UEF also produced significantly greater plant height (at 5–10%) and biomass (at 10%) than UE at the equivalent concentration (*p* < 0.05). In contrast, UE treatments showed no significant difference from WT across all tested concentrations, and when compared with the HO-positive control, both UEF and UE treatments exhibited lower plant height and biomass yield (Figure 2B,C).

Figure 2D,E present the effects of *U. prolifera* extracts on radish root development. Two-way ANOVA showed that root diameter was influenced solely by extract type (*p* < 0.001, Table S2_supplementary data), with no significant concentration or interaction effect. At each concentration, all UEF treatments significantly increased root diameter compared with UE treatments at the equivalent concentration (*p* < 0.05, Figure 2D), with the maximum value (1.7 cm) recorded at 5% UEF. UE extracts had no significant effect on root swelling when compared to WT and HO controls. For root weight, a significant concentration × extract type interaction was detected (*p* < 0.01, Table S2_supplementary data), and a typical hormetic concentration-response relationship was observed under both UE and UEF treatments (Figure 2E). At low concentrations, UEF (5–10%) significantly promoted root growth relative to the WT control (*p* < 0.05) and yielded the maximal underground biomass (5.1 g/plant) at 10%; at these concentrations, UEF also significantly outperformed UE at the equivalent concentration (*p* < 0.05). A similar but less pronounced stimulatory effect was detected at moderate UE concentrations (15–20%, *p* < 0.05), at which radish root weight was comparable to that of the HO control. Nevertheless, this promotion was diminished at lower and higher concentrations (Figure 2E)

### 3.2. Plant Nutritional Quality

**Figure 3:**
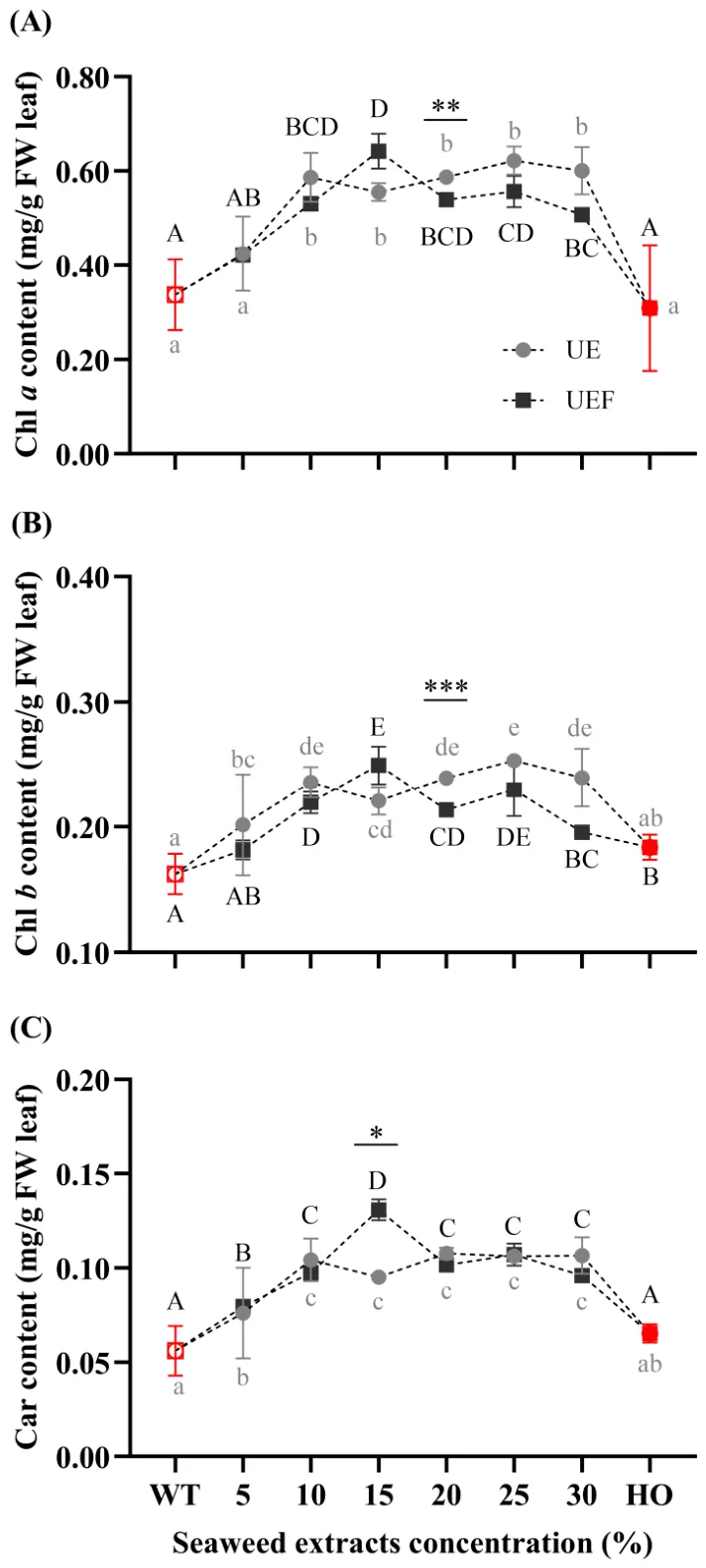
Effects of *U. prolifera* extracts on the leaf pigment content of cherry radish. (**A**) Chl *a* content. (**B**) Chl *b* content. (**C**) Carotenoid content. UE and UEF represent the extracts of *U. prolifera* prepared via enzymatic hydrolysis and coupled enzymatic hydrolysis–microbial fermentation, respectively. Different uppercase letters indicate significant differences among UEF treatments, and different lowercase letters indicate significant differences among UE treatments (Duncan’s test, *p* < 0.05). Asterisks indicate significant differences between UE and UEF at the same concentration (independent-sample *t*-test with Benjamini–Hochberg FDR correction: *, *p* < 0.05; **, *p* < 0.01; ***, *p* < 0.001). WT and HO represent the treatment groups irrigated with pure water (red open circles/squares) and Hoagland nutrient solution (red solid circles/squares), respectively.

Figure 3 shows leaf pigment contents of cherry radish under various treatments. Two-way ANOVA showed that Chl *a* and Chl *b* were significantly affected by extract concentration, extract type, and their interaction (Table S2_supplementary data, *p* < 0.05). In contrast, Car content was significantly affected by concentration and the interaction term, but not by extract type alone (*p* > 0.05). The highest partial η^2^ values (0.587–0.798) identified extract concentration as the primary contributor to pigment variation. As shown in Figure 3, pigment contents increased with UE concentration from 5% to 10% and remained stable thereafter up to 30%; under UEF treatments, all pigments rose from 5% to 15% and then decreased progressively. Three pigments showed similar response patterns to UE and UEF extracts, with pigment levels consistently exceeding those of WT and HO controls (*p* < 0.05). The maximum values of Chl *a*, Chl *b*, and Car (0.64, 0.25, and 0.13 mg/g FW, respectively) were recorded at 15% UEF. At this concentration, UEF yielded significantly higher Car contents than UE (*p* < 0.05), whereas at 20%, UE performed better than UEF in Chl *a* and Chl *b* accumulation (*p* < 0.01).

**Figure 4:**
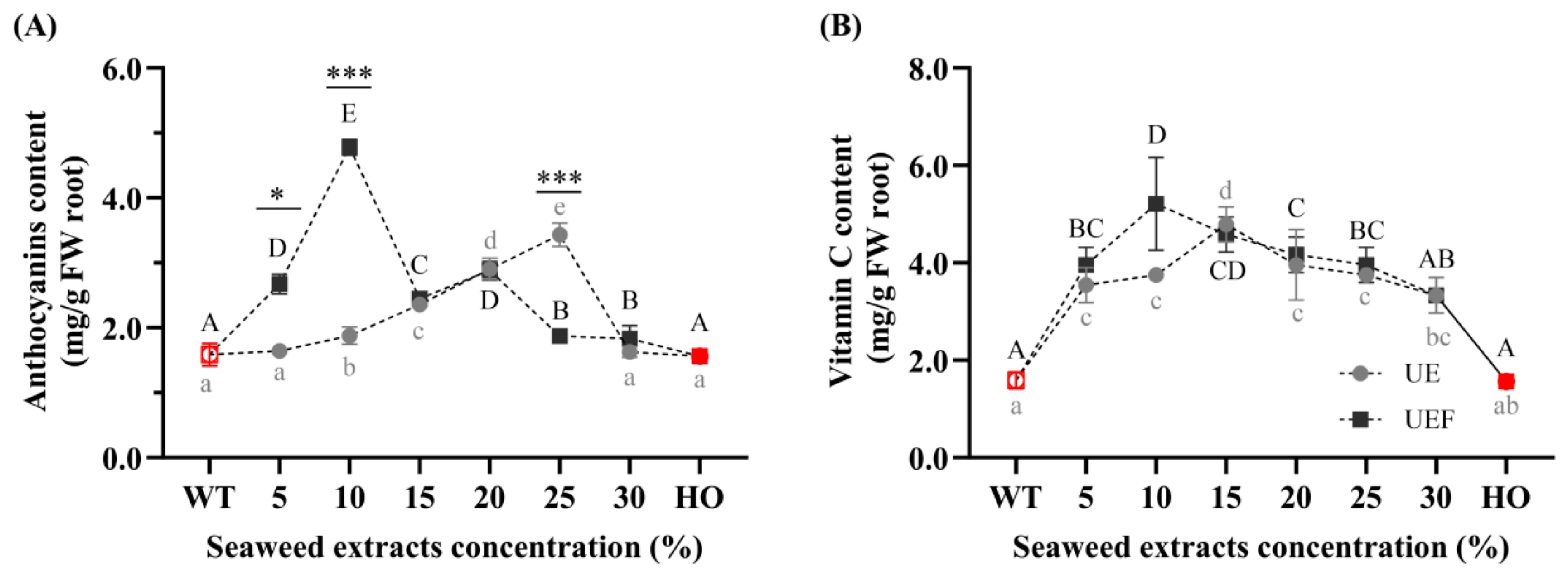
Effects of *U. prolifera* extracts on the root anthocyanin and vitamin C content of cherry radish. (**A**) Anthocyanin content, and (**B**) vitamin C content. UE and UEF represent the extracts of *U. prolifera* prepared via enzymatic hydrolysis and coupled enzymatic hydrolysis–microbial fermentation, respectively. Different uppercase letters indicate significant differences among UEF treatments, and different lowercase letters indicate significant differences among UE treatments (Duncan’s test, *p* < 0.05). Asterisks indicate significant differences between UE and UEF at the same concentration (independent-sample *t*-test with Benjamini–Hochberg FDR correction: *, *p* < 0.01; ***, *p* < 0.001). WT and HO represent the treatment groups irrigated with pure water (red open circles/squares) and Hoagland nutrient solution (red solid circles/squares), respectively.

Figure 4A shows that all UEF treatments markedly enhanced root anthocyanin levels relative to both WT and HO controls (*p* < 0.05). For UE treatments, root anthocyanin content increased progressively as the extract concentration rose from 10% to 25%, whereas no significant enhancement was observed at 30%. The highest anthocyanin contents were recorded at 10% UEF (4.8 mg/g FW) and 25% UE (3.4 mg/g FW). At 5–10% concentrations, UEF treatments exhibited significantly higher anthocyanin contents than UE treatments at equivalent concentrations (*p* < 0.05), whereas at 25%, UE showed a higher level than UEF (*p* < 0.001).

Root vitamin C content was significantly affected by extract concentration (*p* < 0.001) and extract type (*p* < 0.05, Table S2_supplementary data), whereas their interaction showed no significant effect. Under both UE and UEF treatments, vitamin C content exhibited a biphasic response, increasing at lower concentrations and decreasing at higher concentrations, with all treatments exceeding the two control groups (Figure 4B). The maximum values were recorded at 10% UEF (5.2 mg/g FW) and 15% UE (4.8 mg/g FW), respectively.

**Figure 5:**
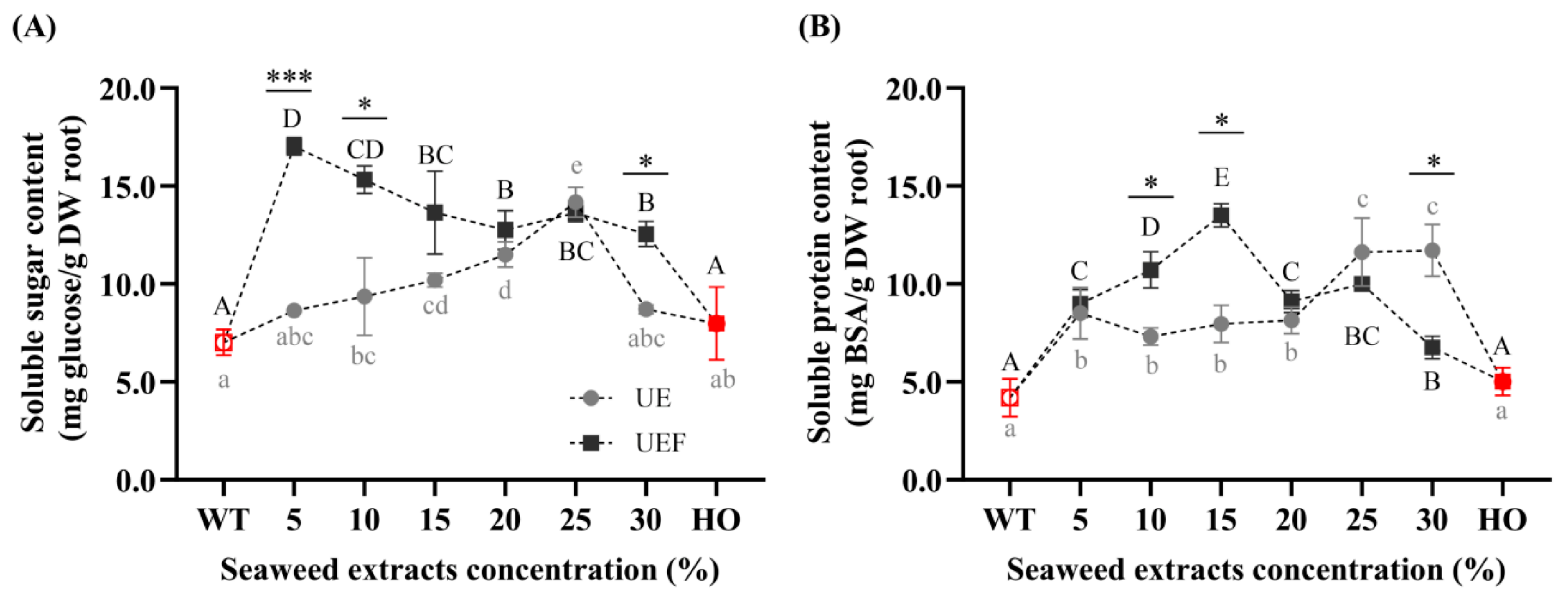
Effects of *U. prolifera* extracts on the root soluble sugar and protein content of cherry radish. (**A**) Soluble protein content and (**B**) soluble carbohydrate content. UE and UEF represent the extracts of *U. prolifera* prepared via enzymatic hydrolysis and coupled enzymatic hydrolysis–microbial fermentation, respectively. Different uppercase letters indicate significant differences among UEF treatments, and different lowercase letters indicate significant differences among UE treatments (Duncan’s test, *p* < 0.05). Asterisks indicate significant differences between UE and UEF at the same concentration (independent-sample *t*-test with Benjamini–Hochberg FDR correction: *, *p* < 0.05; ***, *p* < 0.001). WT and HO represent the treatment groups irrigated with pure water (red open circles/squares) and Hoagland nutrient solution (red solid circles/squares), respectively.

Root soluble sugar content was also significantly affected by extract concentration, extract type, and their interaction (*p* < 0.001, Table S2_supplementary data). Under UE treatments, soluble sugar content increased progressively from 10% to 25%, but returned to levels comparable to those of the WT and HO controls at 30% (*p* > 0.05, Figure 5A). In contrast, all UEF treatments resulted in higher soluble sugar contents than both control groups (*p* < 0.05), with the maximum value recorded at 5% UEF (17.0 mg glucose/g DW), followed by a gradual decline as UEF concentration increased. At 5%, 10% and 30% concentrations, UEF exhibited a greater promoting effect on soluble sugar accumulation than UE (*p* < 0.05).

Two-way ANOVA revealed that root soluble protein content was significantly influenced by extract concentration and its interaction with extract type (*p* < 0.001), while extract type alone showed a small effect size (Partial η^2^ = 0.148, Table S2_supplementary data). All *U. prolifera* extracts significantly enhanced root SP accumulation compared with the WT and HO controls (*p* < 0.05, Figure 5B). Under UEF treatments, SP content increased from 9.0 to 13.5 mg BSA/g DW as the extract concentration rose from 5% to 15%, then declined to 6.7 mg BSA/g DW at 30%. Under UE treatments, SP content remained relatively stable at approximately 8.0 mg BSA/g DW across the 5–20% concentrations, and then increased to 11.7 mg BSA/g DW at 25% and 30%. At equivalent concentrations, UEF exhibited a stronger promoting effect than UE at low concentrations (10% and 15%), whereas UE outperformed UEF at 30% (*p* < 0.05).

### 3.3. Soil Physicochemical Properties

**Figure 6:**
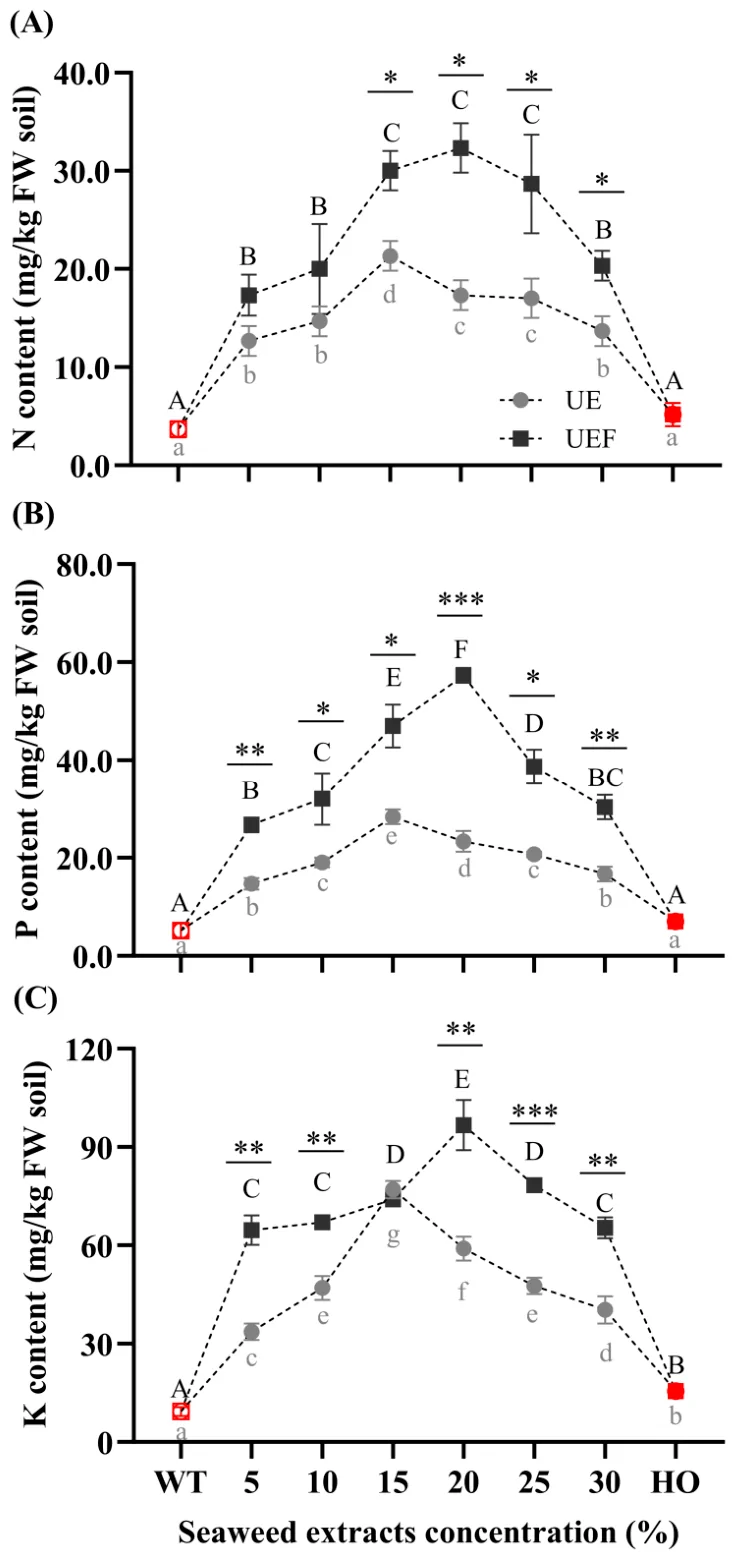
Effects of *U. prolifera* extracts on soil fertility (UE and UEF represent the extracts of *U. prolifera* obtained via enzymatic hydrolysis and coupled enzymatic hydrolysis–microbial fermentation, respectively. (**A**) N content; (**B**) P content; (**C**) K content. Different uppercase letters indicate significant differences among UEF treatments, and different lowercase letters indicate significant differences among UE treatments (Duncan’s test, *p* < 0.05). Asterisks indicate significant differences between UE and UEF at the same concentration (independent-sample *t*-test with Benjamini–Hochberg FDR correction: *, *p* < 0.05; **, *p* < 0.01; ***, *p* < 0.001). WT and HO represent the treatment groups irrigated with pure water (red open circles/squares) and Hoagland nutrient solution (red solid circles/squares), respectively.

Soil N, P, and K contents were significantly influenced by extract concentration, extract type, and their interaction (*p* < 0.001, Table S2_supplementary data). Both extracts induced a similar unimodal response in soil nutrients, with contents rising to a maximum before declining (Figure 6). For UE treatments, peak N, P, and K contents were all recorded at 15% concentration, reaching 21, 28, and 77 mg/kg FW soil, respectively, which represents 4.1-, 4.0-, and 5.0-fold increases over the HO control. For UEF treatments, the maxima were observed at 25% concentration, reaching 32, 57 and 97 mg/kg FW soil, respectively, representing 6.3-, 8.2-, and 6.2-fold increases over the HO control. Overall, UEF enhanced soil nutrients more effectively than UE at equivalent concentrations, with the exception of N at 5–10% and K at 15%.

After 40 days of cultivation, soil EC decreased across all treatments (from 1.66–1.87 mS/cm to ≤517 μS/cm, Table 1). For both UE and UEF treatments, soil EC increased with extract concentration and peaked at 30% concentration. At high concentrations (25–30%), UEF treatments showed higher EC values than UE treatments. Additionally, compared with the pure water control, the HO solution and both UE and UEF extracts significantly decreased soil pH by approximately 0.58 units.

## 4. Discussion

Overuse of chemical fertilizers and pesticides may cause soil degradation, environmental pollution, and biodiversity reduction [34]. Developing green agricultural inputs therefore has become a research priority. Seaweed extracts, rich in diverse bioactive compounds, have been shown to promote crop growth and offer a promising alternative characterized by biodegradability, non-toxicity, and environmental compatibility. Frequent *Ulva* blooms in the Yellow Sea result in massive accumulation of algal biomass, which exacerbates coastal environmental pollution and severely disturbs aquaculture activities [35]. The conversion of waste green-tide biomass into biofertilizers and biostimulants provides a sustainable strategy to mitigate marine ecological degradation. Meanwhile, the agricultural utilization of recycled algal products reduces reliance on synthetic agrochemicals, thereby achieving dual benefits of marine environmental remediation and sustainable crop production.

Our previous study demonstrated the agronomic potential of *U. prolifera*-derived bio-fermentation extract (UF) for cherry radish [15], but it focused on a single preparation process and did not compare different extraction strategies. The present study extends this work by systematically comparing two distinct processes—single enzymatic hydrolysis (UE) and enzyme-coupled microbial fermentation (UEF)—and evaluating their agronomic efficacy across different concentrations. Both extracts exhibited significant concentration-dependent effects on cherry radish growth, nutritional quality, and soil fertility, displaying a typical biphasic “low-promotion, high-inhibition” dose–response pattern. Specifically, compared with the WT and HO controls, both extracts significantly increased the contents of leaf photosynthetic pigments, root anthocyanins, vitamin C, soluble sugars and soluble proteins, as well as soil N, P and K contents at all tested concentrations (Figure 3, Figure 4, Figure 5 and Figure 6), suggesting that *U. prolifera* extracts can improve radish quality and soil fertility. However, all measured indicators exhibited a declining trend once the extract concentration exceeded the optimal threshold. UEF at low concentrations (5–15%) significantly promoted growth, with the most pronounced effects observed at 10% concentration (Figure 2). For UE extract, only the concentration at 15–20% enhanced underground growth (root weight, Figure 2E); either lower or higher concentrations resulted in yield reduction. This typical hormetic biphasic response aligns with previous findings on seaweed extracts in many crops. For instance, shoot length, root length, and dry weight of mung bean all exhibited a characteristic hormetic dose–response when treated with extracts from brown algae (*Ecklonia arborea*, *Gracilaria parvispora*, and *Sargassum horridum*) [36]. Castellanos-Barriga et al. [37] also confirmed that *Ulva lactuca* extract at appropriate concentrations promoted mung bean seedling growth, whereas all growth parameters declined significantly once the concentration exceeded a critical threshold.

The growth-promoting effects of the extracts can be attributed to their richness in phytohormones (auxins, cytokinins, gibberellins, and abscisic acid, etc.), and mineral nutrients (N, P and K) and polysaccharides (Tables S3 and S4_supplementary data), which may synergistically enhance photosynthetic efficiency, activate antioxidant defense systems, and promote root development [1]. Moncada et al. [38] compared the growth-promoting effects of a commercial seaweed extract and the synthetic auxin NAA on lettuce and tomato seedlings, and found that the seaweed extract outperformed NAA at equivalent concentrations in improving seedling quality and shoot/root growth. Zhao et al. [39] also reported that *U. prolifera*-derived polysaccharides (PUP) at an optimal concentration (300 mg/L) increased tobacco fresh weight by 24–70%, with the underlying mechanism involving enhanced synthesis of photosynthetic pigments, including carotenoids, chlorophyll *a*, and chlorophyll *b*.

In contrast, the inhibitory effect at supra-optimal concentrations may be attributable to osmotic stress induced by the high solute load of the extracts, as evidenced by elevated soil EC values (Table 1) under high-concentration treatments [10]. It is well established that excessive accumulation of soluble salts in the rhizosphere can reduce the osmotic potential of the soil solution, thereby limiting water uptake by roots and inducing physiological drought stress [40]. A previous study on sweet pepper seedlings demonstrated that both very high and very low EC treatments significantly decreased plant height, stem diameter, shoot dry weight, and leaf net photosynthetic rate, while increasing malondialdehyde content and antioxidant enzyme activities, indicating that high EC induces abiotic stress through either nutrient deficiency or salinity [41]. In the present study, the elevated soil EC at high extract concentrations likely imposed osmotic stress on cherry radish roots, which may have counteracted the growth-promoting effects of the bioactive compounds. Additionally, excessive concentrations of certain bioactive compounds may exert antagonistic effects or disrupt hormonal homeostasis, thereby impairing normal physiological processes [42]. However, it should be noted that the specific contribution of osmotic stress to the observed growth inhibition was not directly quantified in this study, and this mechanism needs further investigation.

A key finding of this study is the marked difference in growth-promoting efficacy between the two types of extracts. Although both extracts improved cherry radish growth and quality at their respective optimal concentrations, UEF exhibited superior performance in growth traits at low concentrations (5–10%), particularly at 10%. Furthermore, at equivalent concentrations, UEF generally outperformed UE in enhancing root anthocyanins, vitamin C, soluble proteins, soluble sugars, and soil N, P, and K contents. This differential response is fundamentally attributable to the distinct compositional profiles of the two extracts. UEF contained higher levels of several key phytohormones than UE, including cytokinins (N6-(delta2-isopentenyl) adenine: 2.0454 vs. 1.2606 ng/mL; N6-benzyladenine: 0.6684 vs. 0.474 ng/mL), auxin-related compounds (methyl indole-3-acetate: 7.002 vs. 5.0088 ng/mL; N-(3-indolylacetyl)-L-alanine: 9.5394 vs. 7.212 ng/mL), jasmonic acid (3.0852 vs. 2.3358 ng/mL), and salicylic acid 2-O-beta-glucoside (10.3248 vs. 8.2092 ng/mL, Table S3_supplementary data). These compounds can act synergistically to regulate cell division, root initiation, senescence delay, and antioxidant defense. In addition, UEF contained higher levels of phosphorus (237.33 vs. 158.67 mg/L) and potassium (1610 vs. 1180 mg/L) than UE (Table S4_supplementary data)), providing a material basis for biomass accumulation and quality improvement.

The UEF process employed a microbial consortium of *B. amyloliquefaciens*, *L. plantarum*, and *S. cerevisiae*. The three strains may secrete complementary hydrolases (cellulase, hemicellulase, protease, pectinase, amylase, etc.) that synergistically degrade *U. prolifera* cell walls, releasing intracellular components (e.g., sugars, proteins, phytohormones, betaines, phenolic compounds, and polyamines) and depolymerizing macromolecules into low-molecular-weight bioactives (Table S1_supplementary data) [1,43]. In contrast, UE relies solely on cellulase-mediated cell wall disruption, which only partially decomposes algal biomass and releases limited nutrients and bioactive metabolites [44]. Consistent with these findings, Ihua et al. [45] confirmed that microbial enzymatic cocktails derived from macroalgae-degrading consortia exhibit superior efficiency in bioactive compound extraction compared with single commercial enzyme systems, further verifying the technical advantages of enzyme-coupled microbial fermentation over single-enzymatic hydrolysis. Collectively, the coupling of enzymatic hydrolysis with microbial fermentation not only released intracellular bioactive compounds through cell wall disruption but also further transformed and enriched phytohormones and mineral nutrients through fermentation, enabling UEF to achieve superior growth-promoting and quality-enhancing effects at lower concentrations than UE.

The two types of seaweed extracts and their application concentrations differentially regulated soil physicochemical characteristics. Compared with the WT and HO controls, both UE and UEF treatments significantly increased soil available N, P, and K contents (Figure 6). At equivalent concentrations, UEF exhibited a greater improving effect on soil fertility than UE, which may be partly attributable to the nutrient (i.e., P and K) content in UEF itself (Table S4). Notably, although the N contents of the two extracts were similar (357.3 vs. 356.3 mg/L), soil available N was higher under UEF treatment (Figure 6A), suggesting that this difference did not originate from the N content of the extracts themselves. The organic acids and low-molecular-weight carbon sources abundant in UEF may have stimulated rhizosphere microbial activity, thereby accelerating organic N mineralization and increasing soil available N accumulation [46]. Similar mechanisms have been reported for seaweed-derived biostimulants, in which organic components serve as carbon sources that stimulate beneficial rhizosphere microorganisms and improve nutrient cycling [47]. However, direct evidence for these mechanisms was not obtained in this study, and further verification through N form analysis and microbial functional assays is needed.

Soil pH determination showed that compared with the WT control, all extracts (the pH of diluted extracts ranged from 5.4 to 6.0) induced a significant reduction in soil pH by 0.5–0.6 units, which was consistent with the pH decline observed in our previous study using microbially fermented *U. prolifera* extracts [15]. The mild soil acidification could be attributed to inherent organic acids (e.g., alginic acid) in the seaweed extracts, as well as microbial metabolic acids (e.g., lactic acid and humic acid) generated during fermentation. These findings suggest that *U. prolifera* extracts have the potential to improve the physicochemical properties of mildly saline–alkali soil and may offer potential benefits for the management of mildly saline–alkali land. However, although soil EC was reduced in all treatment groups (from 1.66–1.87 mS/cm to ≤517 μS/cm, Table 1), the application concentration of seaweed extract should still be carefully optimized to avoid secondary salinization, given the increase in soil EC at higher extract concentrations.

Notably, after 40 days of incubation, soil pH in all extract-treated groups converged to approximately 7.1 (Table 1), reflecting the strong acid-buffering capacity of the test soil. In such soil, the relatively high initial pH (7.8–8.2) suggests the possible presence of a carbonate-bicarbonate buffering system, which would effectively neutralize the weak organic acids introduced by the extracts [48,49]. Additionally, soil clay minerals and organic matter may also have contributed to pH buffering through cation exchange and protonation/deprotonation reactions [48,50]. Nevertheless, the relative contributions of these buffering mechanisms require further investigation.

## 5. Conclusions

This study demonstrated that both UE and UEF extracts exert significant concentration-dependent effects on cherry radish growth, nutritional quality, and soil fertility, displaying a typical biphasic “low-promotion, high-inhibition” dose–response pattern. The UEF extract, which was prepared via enzymatic hydrolysis coupled with microbial fermentation, exhibited superior agronomic performance at lower concentrations (5–10%) compared to UE (optimal at 15–20%). This superiority can be explained by the efficient release of seaweed-derived bioactive compounds in UEF, which facilitate root development, improve crop nutritional quality, and enhance soil N, P, and K availability. Furthermore, the moderate soil acidification induced by *U. prolifera* extracts showed their positive potential for the amelioration of saline–alkaline soils. These findings suggest that *U. prolifera* extracts, particularly UEF, may serve as effective dual-function biostimulants and soil conditioners, offering a “waste-to-value” solution for green tide management and sustainable agriculture. However, the quantitative links between the functional metabolite profiles of UE and UEF extracts and their agronomic effects, as well as the underlying mechanisms involving gene regulation and rhizosphere microbial community dynamics, remain to be elucidated, which would provide a scientific basis for the functional characterization and precision application of *U. prolifera*-derived biostimulants.

## Figures and Tables

**Table 1:** Effects of *U. prolifera* extracts on the soil pH and electrical conductivity. UE and UEF represent the extracts of *U. prolifera* prepared via enzymatic hydrolysis and coupled enzymatic hydrolysis-microbial fermentation, respectively. Different letters indicate significant differences among UEF treatments, and different lowercase letters indicate significant differences among UE treatments (Duncan’s test, *p* < 0.05). Asterisks indicate significant differences between UE and UEF at the same concentration (independent-sample *t*-test with Benjamini–Hochberg FDR correction: *, *p* < 0.05). WT and HO represent the treatment groups irrigated with pure water and Hoagland nutrient solution, respectively.

Treatments	EC (μS/cm)		pH	
	UE	UEF	UE	UEF
WT	70 ± 5 ^a^	70 ± 5 ^a^	7.63 ± 0.08 ^b^	7.63 ± 0.08 ^b^
5%	363 ± 10 ^cd^	362 ± 11 ^c^	7.10 ± 0.10 ^a^	7.00 ± 0.06 ^a^
10%	350 ± 35 ^c^	400 ± 6 ^d^	7.07 ± 0.06 ^a^	7.07 ± 0.06 ^a^
15%	380 ± 23 ^cde^	422 ± 25 ^de^	7.07 ± 0.06 ^a^	7.03 ± 0.06 ^a^
20%	395 ± 15 ^de^	453 ± 28 ^ef^	7.07 ± 0.06 ^a^	7.07 ± 0.06 ^a^
25%	398 ± 16 ^de^	477 ± 17 ^f^ *	7.07 ± 0.06 ^a^	7.07 ± 0.06 ^a^
30%	409 ± 21 ^e^	517 ± 8 ^g^ *	7.07 ± 0.06 ^a^	7.07 ± 0.06 ^a^
HO	297 ± 41 ^b^	297 ± 41 ^b^	7.10 ± 0.09 ^a^	7.10 ± 0.09 ^a^

## Data Availability

Data available in a publicly accessible repository.

## Supplementary Materials

The following supporting information can be downloaded at: https://www.mdpi.com/article/10.3390/plants15192978/s1. Table S1. Strain selection rationale and potential functional roles in *Ulva prolifera* fermentation. Table S2. Two-way ANOVA analyses assessing interactive effects between concentration and preparation process of *Ulva prolifera* extracts on cherry radish growth traits (plant height, plant weight, root diameter, root weight), leaf pigment contents, root anthocyanin, vitamin C, soluble sugar, soluble protein, soil N, P and K concentrations, soil pH and EC. Table S3. Contents of phytohormones in the undiluted seaweed extracts. Table S4. Contents of nitrogen, phosphorus, potassium and polysaccharides in undiluted seaweed extract [51,52,53,54,55,56,57,58,59,60,61,62].
