# Supplementary material for: Valorization of Ulva prolifera Biomass into Biostimulants: Effects of Extraction Processes on Cherry Radish Growth, Nutritional Quality and Soil Fertility
Source: Plants (Basel). 2026 Sep 29;15(19):2978. doi: 10.3390/plants15192978 (PMC13666734; doi:10.3390/plants15192978)
Supplement: Supplementary file 1 [file plants-15-02978-s001.zip › plants-4570483-supplementary.pdf]

Supplementary data for the article:

Valorization of *Ulva prolifera* biomass into biostimulants: effects of extraction processes on cherry radish yield, nutritional quality and soil fertility

Hailong Wu[1], Wenli Jiang[1], Wen Liu[1] He Li[1], Sufang Li[1,2*], Fei Xu[3], Fengrong Tian[4], Xinxing Shi[4], Baolin Yuan[5]

[1]Jiangsu Key Laboratory of Marine Bioresources and Environment, Jiangsu Institute of Marine Resources Development, Jiangsu Ocean University, Lianyungang 222005, China

[2]State Key Laboratory of Marine Environmental Science, College of Ocean and Earth Sciences, Xiamen University, Xiamen 361005, China

[3]Jiangsu Zhonglian Agricultural Development Co., Ltd, Lianyungang 222023, China

[4]China Zhonglan Lianhai Design and Research Institute Co., Ltd., Lianyungang 222004, China

[5]Lianyungang Agricultural and Commercial Holding Group Co., Ltd., Lianyungang 222006, China

** Corresponding author*

E-mail address:lisuf@jou.edu.cn (S. Li)

Materials and methods

Chemical characterization of seaweed extracts

The phytohormone contents in the undiluted UE and UEF extracts were determined by ultra-performance liquid chromatography-tandem mass spectrometry (UPLC-MS/MS) according to the method of Yalçın et al. [51], with minor modifications. Briefly, an aliquot of each extract was mixed with methanol-water (75:25, v/v), ultrasonicated for 10 min, vortexed for 2 min, and extracted at −20 °C for 16 h. After extraction, the mixture was centrifuged at 5000 rpm for 10 min, and the residue was re-extracted with additional solvent for 1 h. The supernatants were combined, filtered, and evaporated to dryness under vacuum. The dried extract was redissolved in 20% methanol solution adjusted to pH 3.0 with formic acid, and purified using an OASIS HLB solid-phase extraction cartridge (3 cc, 60 mg; Waters, Milford, MA, USA). The cartridge was preconditioned with methanol, ultrapure water, and 20% methanol solution (pH 3.0), respectively. After loading the sample, the cartridge was washed with 2 mL of water and eluted with 2 mL of methanol. The eluate was filtered through a 0.45 μm membrane and analyzed by UPLC-MS/MS.

Chromatographic separation was performed on a reversed-phase C18 column with a gradient elution of 0.01% formic acid in water (A) and 0.01% formic acid in acetonitrile (B). The phytohormones were detected in multiple reaction monitoring (MRM) mode using an electrospray ionization (ESI) source, with IAA, BAP, and zeatin analyzed in positive ion mode, and kinetin, GA, and ABA analyzed in negative ion mode. Quantification was performed using the external standard method, and the results were expressed as ng/mL. The phytohormone standards, including N6-(delta2-isopentenyl) adenine, N6-benzyladenine, dihydrozeatin riboside, methyl indole-3-acetate, N-(3-indolylacetyl)-L-alanine, indole-3-carboxaldehyde, gibberellic acid A3, jasmonic acid, salicylic acid 2-O-beta-glucoside, and abscisic acid, were purchased from Sigma-Aldrich (St. Louis, MO, USA) or Olchemim Ltd. (Olomouc, Czech Republic).

The polysaccharide content of the extracts was determined using the phenol-sulfuric

acid method as described by DuBois et al. [52], with glucose as the standard. Briefly, an aliquot of each extract was mixed with 5% phenol solution and concentrated sulfuric acid, heated at 100 °C for 15 min, and cooled to room temperature. The absorbance was measured at 490 nm using a UV-1800 spectrophotometer (Shimadzu, Japan), and the polysaccharide content was calculated from a glucose standard curve and expressed as mg/mL.

The N, P, and K contents of the undiluted extracts were determined using commercial assay kits (Nanjing Jiancheng Bioengineering Institute, Nanjing, China) according to the manufacturer's instructions.

Table S1. Strain selection rationale and potential functional roles in *Ulva prolifera* fermentation

Microorganism	Enzymatic Profile	Potential Roles in Fermentation	Reference
Bacillus amyloliquefaciens	Broad hydrolases (cellulase, hemicellulase, pectinase, protease, amylase)	Degrades *U. prolifera* cell wall; facilitates release of intracellular bioactives (proteins, polysaccharides, vitamins, phytohormones, minerals); depolymerizes macromolecules into smaller bioactive compounds	[53–56]
Lactobacillus plantarum	Protease, cellulase, hemicellulase, glycosidase, amylase, lipase	Disrupts cell wall; converts complex biomolecules into sugars, peptides, amino acids, and organic acids (e.g., lactic acid, SCFAs)	[55,57–60]
Saccharomyces cerevisiae	Multienzyme (cellulase, pectinase, xylanase, mannanase, amylase, glycosidase, lipase, protease, invertase)	Contributes to cell wall disruption; converts macromolecules into crop-beneficial active compounds	[55,61,62]

Table S2. Two-way ANOVA analyses assessing interactive effects between concentration and preparation process of *Ulva prolifera* extracts on cherry radish growth traits (plant height, plant weight, root diameter, root weight), leaf pigment contents, root anthocyanin, vitamin C, soluble sugar, soluble protein, soil N, P and K concentrations, soil pH and EC.

Factors	df	F	*P*	Partial η^2
**Plant height**				
Concentration	5	6.299	0.001	0.568
Extract type	1	13.257	0.001	0.356
Concentration × Extract type	5	3.566	0.015	0.426
**Plant weight**				
Concentration	5	4.924	0.003	0.506
Extract type	1	4.857	0.037	0.168
Concentration × Extract type	5	2.757	0.042	0.365
**Root diameter**				
Concentration	5	1.15	0.366	0.215
Extract type	1	72.448	0.000	0.775
Concentration × Extract type	5	0.937	0.477	0.182
**Root weight**				
Concentration	5	4.954	0.007	0.623
Extract type	1	3.374	0.086	0.184
Concentration × Extract type	5	6.086	0.003	0.67
**Leaf Chl *a* content**				
Concentration	5	18.947	0.000	0.798
Extract type	1	6.331	0.019	0.209
Concentration × Extract type	5	4.837	0.003	0.502
**Leaf Chl *b* content**				
Concentration	5	6.821	0.000	0.587
Extract type	1	9.394	0.005	0.281
Concentration × Extract type	5	3.237	0.023	0.403
**Leaf Car content**				
Concentration	5	11.67	0.000	0.709
Extract type	1	0.926	0.345	0.037
Concentration × Extract type	5	5.811	0.001	0.548
**Root anthocyanins content**				
Concentration	5	115.389	0.000	0.96
Extract type	1	106.565	0.000	0.816
Concentration × Extract type	5	197.06	0.000	0.976
**Root vitamin C content**				
Concentration	5	7.158	0.000	0.599
Extract type	1	5.263	0.031	0.18

Concentration × Extract type	5	2.484	0.06	0.341
**Soluble sugar content**				
Concentration	5	7.033	0.000	0.594
Extract type	1	125.384	0.000	0.839
Concentration × Extract type	5	15.427	0.000	0.763
**Soluble protein content**				
Concentration	5	6.738	0.000	0.584
Extract type	1	4.166	0.052	0.148
Concentration × Extract type	5	23.591	0.000	0.831
**Soil N content**				
Concentration	5	18.852	0.000	0.797
Extract type	1	102.684	0.000	0.811
Concentration × Extract type	5	3.656	0.013	0.432
**Soil P content**				
Concentration	5	55.158	0.000	0.92
Extract type	1	437.333	0.000	0.948
Concentration × Extract type	5	14.748	0.000	0.754
**Soil K content**				
Concentration	5	62.227	0.000	0.928
Extract type	1	366.89	0.000	0.939
Concentration × Extract type	5	22.593	0.000	0.825
**EC**				
Concentration	5	22.488	0.000	0.824
Extract type	1	71.475	0.000	0.749
Concentration × Extract type	5	5.104	0.003	0.515
**pH**				
Concentration	5	0.3	0.908	0.059
Extract type	1	1.786	0.194	0.069
Concentration × Extract type	5	1.1	0.386	0.186

Table S3. Contents of phytohormones in the undiluted seaweed extracts.

Seaweed extracts	N6-(delta2-Isopentenyl) adenine (ng/mL)	N6-benzyladenine (ng/mL)	Dihydrozeatin riboside (ng/mL)	Methyl indole-3-acetate (ng/mL)	N-(3-Indolylacetyl)-L-alanine (ng/mL)	Indole-3-carboxaldehyde (ng/mL)	Gibberellic A3 (ng/mL)	Jasmonic Acid (ng/mL)	SalicylicAcid2-O-beta-Glucoside (ng/mL)	Abscisic Acid (ng/mL)
UE	1.2606	0.474	0.0762	5.0088	7.212	30.3402	2.2482	2.3358	8.2092	5.706
UEF	2.0454	0.6684	0.762	7.002	9.5394	30.1926	2.178	3.0852	10.3248	5.2296

Table S4. Contents of nitrogen, phosphorus, potassium and polysaccharides in undiluted seaweed extract.

Seaweed extracts	N (mg/L)	P (mg/L)	K (mg/L)	Polysaccharides (mg/mL)
HO	210.1	30.97	283	-
UF	356.3	158.67	1180	0.3201
UEF	357.3	237.33	1610	0.3158
